# Household-level surrounding greenspace as a nature-based intervention for health recovery after occupational injury

**DOI:** 10.3389/fpubh.2026.1817203

**Published:** 2026-05-01

**Authors:** Doyun Song, Sieon Kim, Minseo Park, Gaeun Kwon, Choong-hee Park, Changhyou Kim, Jeongho Choi, Jaehoon Kim, Geonwoo Kim

**Affiliations:** 1 Department of Forest Environmental Resources, College of Agriculture and Life Sciences, Gyeongsang National University, Jinju, Republic of Korea; 2 Forest Welfare R&D Center, Korea Forest Welfare Institute (FoWI), Daejeon, Republic of Korea; 3 Forest Policy Division, Korea Forest Service, Daejeon, Republic of Korea

**Keywords:** household-level rehabilitation, nature-based intervention, occupational injury recovery, resilience, urban greenspace

## Abstract

**Introduction:**

Urban greening has been increasingly discussed as a component of health-supportive environments. While greenspace exposure has been associated with psychological restoration, limited evidence exists regarding household-level responses among families affected by occupational injury. This study examined whether a structured forest-based intervention was associated with short-term changes in psychological burden, resilience, and perceived family relationship quality across household roles.

**Methods:**

A single-group pre–post design was applied to injured workers and their cohabiting family members participating in a forest-based rehabilitation program in South Korea. Paired analyses included 1,194 participants. Role-stratified linear mixed-effects models (*N* = 918) adjusted for age and sex. Exploratory heterogeneity analyses focused on resilience (*N* = 789). Outcomes included occupational injury distress, resilience (KRQ-27), and family relationship quality. Mean changes, 95% confidence intervals, Holm-adjusted *p*-values, and paired Hedges’ *g* were reported. Psychometric outcomes were assessed immediately before program initiation and immediately after completion of the final scheduled session.

**Results:**

Occupational injury distress decreased (*Δ* = −7.81, *g* = −0.397, *p* < 0.001), resilience increased (*Δ* = +8.51, *g* = 0.517, *p* < 0.001), and family relationship quality improved over the immediate post-program period (*Δ* = +4.38, *g* = 0.391, *p* < 0.001). Reductions in distress were numerically larger among injured workers, whereas gains in resilience appeared numerically larger among children and spouses. Pre–post changes were observed across income and age strata, although some subgroup contrasts did not remain statistically significant after multiplicity adjustment.

**Discussion:**

Forest-based programming within an urban greenspace context was associated with short-term favorable changes in psychological and relational outcomes. Because the design lacked a control group, outcomes were assessed only within an immediate post-program window, and long-term follow-up was not available, the observed changes should be interpreted as short-term associations rather than confirmed causal effects.

## 1 Introduction

Rapid urbanization has reshaped everyday environmental exposures, often reducing routine contact with natural elements while increasing exposure to chronic psychosocial stress (1). In response, urban greening initiatives—such as expanding parks, urban forests, and accessible neighborhood greenspaces—have increasingly been discussed as components of health-supportive urban environments (2). Although greenspace cannot substitute for clinical treatment, accumulating evidence suggests that contact with natural settings may contribute to psychological restoration, stress reduction, and improved emotional regulation (3). For this reason, greening strategies are gradually being considered as potential complements to conventional public-health and welfare approaches (4, 5).

However, access to high-quality urban greenspace is not evenly distributed. Socioeconomic disadvantage, residential density, and environmental inequality may influence both baseline psychological burden and opportunities for restorative environmental exposure (6, 7). Some research has suggested that individuals experiencing higher levels of stress may demonstrate greater short-term improvements following greenspace exposure, leading to the proposal that greenspace may function as an “equigenic” resource (8, 9). At the same time, these patterns are not universal, and the extent to which greenspace exposure moderates health disparities likely depends on contextual factors such as accessibility, environmental quality, and prior exposure patterns (10, 11).

The potential benefits of greenspace exposure are often attributed to mechanisms related to attention restoration and autonomic regulation. Structured greenspace-based programs typically combine guided walking, paced breathing, relaxation practices, and sensory engagement with natural environments (2). Theoretical accounts propose that such activities may facilitate parasympathetic activation and emotional regulation, potentially contributing to reductions in negative affect (12, 13). Systematic reviews have reported short-term improvements in stress, anxiety, and depressive symptoms following greenspace exposure, along with modest changes in physiological indicators such as heart rate variability (14, 15). Although findings remain heterogeneous, these results may suggest that structured environmental exposure offers supportive benefits for individuals experiencing sustained psychosocial strain.

Occupational injury is commonly managed as an individual medical and vocational condition affecting the injured worker. In practice, its impact often extends to the broader household. Occupational injury can place considerable strain not only on injured workers but also on their families (16). Injured workers frequently report symptoms such as depression, anxiety, anger dysregulation, somatization, and sleep disturbance, which may persist beyond the acute recovery phase (17). Such psychological strain has been associated with delayed return to work and diminished quality of life. Importantly, the impact of injury is rarely confined to the worker (16). Spouses and partners may experience intensified caregiving responsibilities and financial stress (18, 19), while children may face adjustment challenges (20, 21). Nevertheless, current rehabilitation and return-to-work provisions predominantly concentrate on the injured worker (22). By contrast, key family-level impacts, including spousal psychological distress, caregiver burden, and children’s adjustment, are seldom systematically assessed and rarely addressed within the same structured rehabilitation program (18, 23).

The importance of greenspace as a supportive resource was highlighted during the COVID-19 pandemic (24, 25), when public green areas were frequently used as accessible spaces for psychological relief, especially among households without private outdoor environments (26, 27). In South Korea, structured greenspace-based programs have been incorporated into selected public welfare and rehabilitation initiatives (28). Programs delivered in collaboration with the Korea Workers’ Compensation & Welfare Service (K-COMWEL) and forest-welfare institutions provide short-stay or residential greenspace exposure to injured workers and their cohabiting family members to support psychological stabilization and family functioning during recovery.

Despite increasing interest in greening strategies as potential contributors to human health, the mechanisms through which forest-based programs may affect households experiencing occupational injury require clearer conceptualization. Prior research suggests that exposure to natural environments may contribute to psychophysiological stress reduction, restoration of directed attention, improvement in affective state, and opportunities for supportive social interaction (29, 30). For households coping with occupational injury, these processes may be especially relevant because recovery often involves not only individual distress in the injured worker, but also caregiving burden, uncertainty, and relational strain among cohabiting family members (31). Accordingly, forest-based programming may provide a structured, low-burden context in which short-term psychosocial relief and relational improvement become more achievable.

The present study examined pre–post changes in psychological burden, resilience, and perceived family relationship quality following participation in a household-level greenspace intervention among families affected by occupational injury. Using linear mixed-effects models, we estimated role-specific pre–post changes and explored potential heterogeneity across selected sociodemographic factors. Our aim was to characterize short-term associations between program participation and recovery-related outcomes across different family members, rather than to infer confirmed causal effects.

## 2 Materials and methods

### 2.1 Participants and recruitment

Participants were industrial-accident workers (“injured workers”) covered by the national workers’ compensation insurance system who were either receiving medical treatment or had completed treatment within the previous 3 years, as well as their cohabiting family members (spouses/partners, children, or other cohabiting relatives). Participants were recruited through a rehabilitation program administered by the Korea Workers’ Compensation & Welfare Service (K-COMWEL). Program participants were informed about the study and enrolled if they provided written consent. The greenspace-based intervention was delivered by the Korea Forest Welfare Institute (FoWI) as part of the rehabilitation program.

Eligibility required that injured workers had completed acute medical treatment or were undergoing rehabilitation, and that family members participated voluntarily. Exclusion criteria included inability to ambulate safely on forest trails, acute psychiatric instability, and cognitive impairment precluding informed consent. For minors, written parental consent and participant assent were obtained. All procedures complied with institutional and national ethical standards. The study was approved by the Public Institutional Review Board designated by the Ministry of Health and Welfare (Approval No. P01-202512-01-039). Written informed consent was obtained from all participants.

### 2.2 Intervention description

The intervention consisted of structured exposure to designated therapeutic forest environments delivered by nationally certified forest therapy practitioners across participating forest welfare facilities. The standardized module included low-intensity walking, paced breathing, guided relaxation, attentional practices, and nature contemplation, with activities designed to maintain low-to-moderate intensity (approximately 2.0–3.5 METs) to ensure safety within a rehabilitation context. Programs were offered in one-day, 1-night/2-day, and 2-night/3-day formats between April and November 2025. However, the intervention schedule represented in the present analysis was primarily based on a structured 1-night/2-day format, comprising approximately 8 h of forest-based programming. Each session accommodated up to 30 participants, depending on site logistics.

Within this format, the program included orientation, baseline assessment, guided forest-based educational activities, light physical activity, family-oriented interpretation and experience-based sessions, institution-specific regional linkage activities, and immediate post-program assessment. On Day 1, participants completed orientation and the pre-program effectiveness assessment before engaging in guided activities such as forest mission orienteering and light physical activity. On Day 2, participants participated in family-oriented forest interpretation, experience, and play activities, followed by institution-specific specialized programming and the post-program effectiveness assessment. Session length, number of guided activities, attendance, adverse events, and withdrawals were recorded, and facilitators used a session checklist to monitor adherence to the standardized protocol. A detailed example of the program schedule is provided in Supplementary Table 3. Although minor site-level variation in timing or region-linked activities may have occurred, the overall sequence of orientation, baseline assessment, structured forest-based activities, and immediate post-program assessment was maintained across sites. While the intervention protocol was standardized and monitored using session checklists, detailed site-level ecological characteristics (e.g., forest type or composition) and quantitative adherence metrics were not available in the de-identified analytic dataset; therefore, some implementation heterogeneity across facilities may remain.

Psychometric outcomes were assessed twice during the program according to a standardized pre–post sequence. Baseline assessment was conducted on site immediately before the intervention began, typically following orientation on Day 1, whereas post-program assessment was conducted on site immediately after completion of the final scheduled session on Day 2. According to the study procedure, the assessment process, including study explanation, instructions, and questionnaire completion, was designed to be completed within approximately 1 h. Accordingly, the present analyses should be interpreted as reflecting immediate pre–post changes observed within a relatively brief assessment window, rather than sustained effects confirmed through longer-term follow-up.

### 2.3 Outcomes

The primary outcome measure of psychological burden was assessed using the occupational injury distress (Multidimensional Psychological Assessment for Injured Workers–Short Form), a 25-item instrument developed by the Korea Workers’ Compensation & Welfare Service (K-COMWEL) for use with occupationally injured workers and their families. Items are rated on a 5-point Likert scale (32). The scale comprises five 5-item subscales: Anger, Anxiety, Depression, Somatization, and Lack of Social Support. Subscale scores and a total score (range: 25–125) were obtained by summing item responses, with higher scores indicating greater symptom burden. Previous validation research confirmed satisfactory construct validity and internal consistency in occupational injury populations (32). The instrument was administered without modification of item wording or scoring at both pre- and post-intervention assessments. Cronbach’s *α* coefficients for the current sample are presented in Supplementary Table 2.

The resilience measure consisted of 27 items rated on a 5-point Likert scale, comprising three 9-item subscales: Self-Regulation (Self-control), Interpersonal Competence (Sociability), and Positivity (Optimism). Subscale and total scores were derived by summing responses, with higher values denoting greater resilience. The instrument was initially developed by (33) and later adapted for the Korean context (34). Prior validation studies have reported satisfactory reliability (34), and Cronbach’s *α* coefficients for the current sample are presented in Supplementary Table 2.

The family relationship quality measure comprised 15 items rated on a 5-point Likert scale, assessing two domains: Emotional Support and Acceptance and Respect. Subscale and total scores were computed by summing item responses, with higher scores indicating better perceived family relationship quality. Previous research has reported high internal consistency (Cronbach’s *α* = 0.93) (35). Reliability coefficients for the current sample are provided in Supplementary Table 2.

### 2.4 Statistical analysis

All statistical analyses were conducted in R (version 4.4.3). Analyses were performed in three sequential steps to describe program-associated changes and to explore potential differences across family members and sociodemographic groups. First, paired pre–post analyses were conducted for all participants with complete paired data (*N* = 1,194; Table 1). These analyses were performed separately for the three outcome instruments: occupational injury distress (Short Form of the Multidimensional Psychological Test for Injured Workers), resilience (KRQ-27), and family relationship quality. Mean differences between pre- and post-intervention scores were evaluated using paired *t*-tests under the assumption of approximate normality of paired differences. Wilcoxon signed-rank tests were additionally performed as a distribution-free sensitivity analysis. For each scale and subscale, mean change with 95% confidence intervals and corresponding *p*-values were reported. Effect sizes were quantified using paired Hedges’ *g* with small-sample correction to describe the magnitude of pre–post change. Internal consistency (Cronbach’s *α*) was calculated for each instrument at each time point, and basic data quality checks were conducted.

**Table 1:** Paired *t*-test for K-COMWEL, resilience, family relationship (*N* = 1,194).

Domain	Scale	Before (mean ± SD)	After (mean ± SD)	Diff	95% CI	*t*	*p*	Hedges’ *g*	Shapiro–Wilk *p*
Occupational injury distress	Anger	12.750 ± 4.423	10.907 ± 4.107	−1.843	[−2.064, −1.621]	−16.317	<0.001	−0.431	<0.001
	Anxiety	12.190 ± 4.538	10.605 ± 4.122	−1.585	[−1.811, −1.360]	−13.805	<0.001	−0.364	<0.001
	Depression	12.066 ± 4.604	10.415 ± 4.186	−1.651	[−1.874, −1.427]	−14.485	<0.001	−0.374	<0.001
	Somatization	12.415 ± 4.716	10.843 ± 4.172	−1.572	[−1.818, −1.326]	−12.545	<0.001	−0.351	<0.001
	Lack of social support	11.099 ± 4.309	9.938 ± 4.019	−1.161	[−1.379, −0.943]	−10.428	<0.001	−0.278	<0.001
	Total	60.513 ± 20.291	52.706 ± 18.96	−7.807	[−8.787, −6.826]	−15.619	<0.001	−0.397	<0.001
Resilience	Self-control	32.529 ± 5.862	35.251 ± 5.733	2.722	[2.398, 3.046]	16.479	<0.001	0.469	<0.001
	Sociability	31.497 ± 5.623	34.173 ± 5.824	2.676	[2.361, 2.991]	16.655	<0.001	0.467	<0.001
	Optimism	31.345 ± 6.145	34.456 ± 6.254	3.111	[2.774, 3.447]	18.131	<0.001	0.501	<0.001
	Total	95.372 ± 16.111	103.880 ± 16.76	8.508	[7.608, 9.409]	18.541	<0.001	0.517	<0.001
Family relationship	Emotional support	37.291 ± 7.745	40.323 ± 7.52	3.032	[2.62, 3.445]	14.416	<0.001	0.397	<0.001
	Acceptance & respect	19.312 ± 4.001	20.655 ± 3.84	1.343	[1.126, 1.561]	12.107	<0.001	0.342	<0.001
	Total	56.602 ± 11.32	60.978 ± 11.05	4.376	[3.775, 4.977]	14.283	<0.001	0.391	<0.001

*N* = 1,194. Values are mean ± SD. Diff = After − Before. CI = 95% confidence interval for the mean difference. ^***^p < 0.001, ^**^p < 0.01, ^*^p < 0.05, ns.

Second, role-specific linear mixed-effects models were fitted among injured workers, spouses, and children (*N* = 918; Table 2). These models accounted for repeated measurements within individuals by including a subject-specific random intercept. Time (before vs. after), family role, and their interaction were included as fixed effects, with adjustment for sex and mean-centered age. Adjusted marginal means were estimated for each role and time point, and within-role pre–post contrasts were calculated. Multiplicity was controlled using the Holm procedure within each outcome scale. These analyses were conducted to assess whether patterns of change differed across family members.

**Table 2:** Adjusted pre-post results by family members (LMM adjusted for age, total *N* = 918).

Scale	Group (*N*)	EMM (before)	EMM (after)	Diff	95% CI	*p* (Holm)	SMD
Occupational injury distress	Worker (293)	61.565 ± 1.694	52.35 ± 1.694	−9.215	[−11.184, −7.246]	<0.001	−0.498
	Spouse (373)	57.742 ± 1.995	50.077 ± 1.995	−7.665	[−9.410, −5.920]	<0.001	−0.457
	Child (252)	53.377 ± 1.562	46.861 ± 1.562	−6.516	[−8.639, −4.393]	<0.001	−0.405
Resilience	Worker (293)	94.983 ± 1.409	103.41 ± 1.409	8.427	[6.610, 10.243]	<0.001	0.461
	Spouse (373)	96.179 ± 1.651	105.064 ± 1.651	8.885	[7.275, 10.495]	<0.001	0.582
	Child (252)	97.404 ± 1.304	107.328 ± 1.304	9.924	[7.966, 11.884]	<0.001	0.736
Family relationship	Worker (293)	59.15 ± 0.972	62.904 ± 0.972	3.754	[2.544, 4.964]	<0.001	0.333
	Spouse (373)	56.953 ± 1.142	61.586 ± 1.142	4.633	[3.560, 5.705]	<0.001	0.435
	Child (252)	57.639 ± 0.899	61.932 ± 0.899	4.293	[2.989, 5.598]	<0.001	0.453

Values are mean ± SE. Diff = After − Before. CI = 95% confidence interval for the mean difference Estimated marginal means (EMMs) were obtained using the emmeans package in R. SMD (standardized mean difference) was computed as *Δ*EMM/*σ*_residual, where *Δ*EMM denotes the model-estimated change in EMMs and *σ*_residual denotes the residual standard deviation used as the standardizing quantity. ^***^p < 0.001, ^**^p < 0.01, ^*^p < 0.05, ns = not significant.

**Figure 1:**
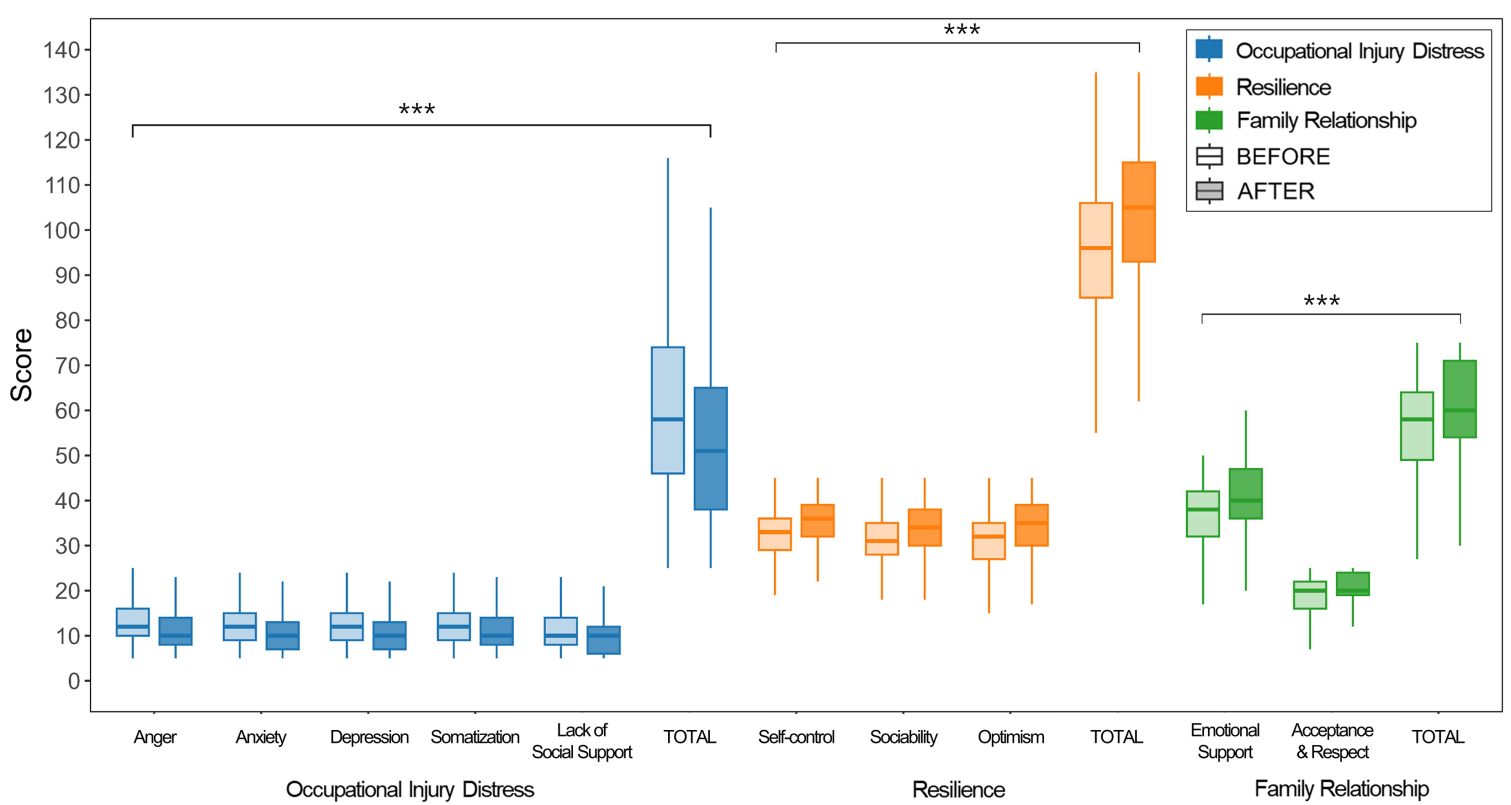
Pre-post distributions.

Third, exploratory heterogeneity analyses were conducted among participants with complete data on age group, forest visit frequency, and household income (*N* = 789; Table 3, Figure 1). Mixed-effects models including interactions with time were fitted, and post–pre contrasts were estimated using model-based marginal means. Holm adjustment was applied to account for multiple comparisons. Given the exploratory nature of these analyses, findings were interpreted cautiously.

**Table 3:** Linear mixed-model estimates of pre-post change by visit × age, income × age, and income × visit in resilience.

Scale	Factor combination (*N*)	Diff	95% CI	*p* (Holm)	SMD
Forest visit frequency × age (789)	Frequent × adolescents and emerging adults (8)	13.125	[2.003, 24.247]	0.029	0.638
	Frequent × adults (95)	6.747	[3.520, 9.975]	<0.001	0.372
	Frequent × older adults (50)	5.540	[1.091, 9.989]	0.029	0.313
	Occasional × adolescents and emerging adults (58)	11.259	[7.128, 15.389]	<0.001	0.816
	Occasional × adults (216)	10.241	[8.100, 12.381]	<0.001	0.670
	Occasional × older adults (86)	8.802	[5.410, 12.194]	<0.001	0.522
	Rare × adolescents and emerging adults (59)	10.831	[6.735, 14.926]	<0.001	0.790
	Rare × adults (170)	8.947	[6.534, 11.360]	<0.001	0.575
	Rare × older adults (47)	7.043	[2.454, 11.631]	0.008	0.405
Monthly household income × age (789)	Low × adolescents and emerging adults (34)	12.824	[7.435, 18.212]	<0.001	0.840
	Low × adults (97)	11.227	[8.036, 14.417]	<0.001	0.671
	Low × older adults (105)	8.829	[5.762, 11.895]	<0.001	0.509
	Middle × adolescents and emerging adults (59)	10.424	[6.333, 14.515]	<0.001	0.743
	Middle × adults (245)	8.910	[6.903, 10.918]	<0.001	0.526
	Middle × older adults (85)	6.631	[2.733, 10.528]	0.002	0.376
	High × adolescents and emerging adults (32)	10.813	[5.258, 16.367]	<0.001	0.809
	High × adults (139)	7.928	[5.263, 10.593]	<0.001	0.585
	High × older adults (13)	0.538	[−8.177, 9.253]	0.903	0.045
Monthly household income × forest visit frequency (789)	Low × frequent (53)	9.623	[5.302, 13.943]	<0.001	0.530
	Low × occasional (101)	11.158	[8.029, 14.288]	<0.001	0.598
	Low × rare (82)	9.939	[6.465, 13.413]	<0.001	0.747
	Middle × frequent (61)	5.426	[1.399, 9.454]	0.017	0.255
	Middle × occasional (180)	9.933	[7.589, 12.278]	<0.001	0.685
	Middle × rare (128)	8.672	[5.892, 11.452]	<0.001	0.515
	High × frequent (39)	4.667	[−0.370, 9.703]	0.069	0.421
	High × occasional (79)	8.949	[5.410, 12.488]	<0.001	0.700
	High × rare (66)	8.576	[4.704, 12.448]	<0.001	0.552

*N* = 789. Estimates are from linear mixed-effects models (LMMs) with a subject-level random intercept and a time × factor1 × factor2 interaction. Diff = post − pre. CI = 95% confidence interval for the estimated mean difference. Estimated marginal means (EMMs) were obtained using the emmeans package in R. SMD (standardized mean difference) was computed as *Δ*EMM/*σ*_residual, where *Δ*EMM denotes the model-estimated change in EMMs and *σ*_residual denotes the residual standard deviation used as the standardizing quantity. ^***^p < 0.001, ^**^p < 0.01, ^*^p < 0.05, ns = not significant.

Across analyses, results were presented as estimates with 95% confidence intervals where applicable. Estimated marginal means (EMMs) were obtained using the emmeans package in R (36), and standardized mean differences (SMDs) for the mixed-effects models were computed from the model-estimated marginal means using the residual standard deviation as the standardizing quantity. The analytical approach was intended to characterize observed patterns of change and should not be interpreted as establishing causal effects.

## 3 Results

### 3.1 Participants and baseline characteristics

**Figure 2:**
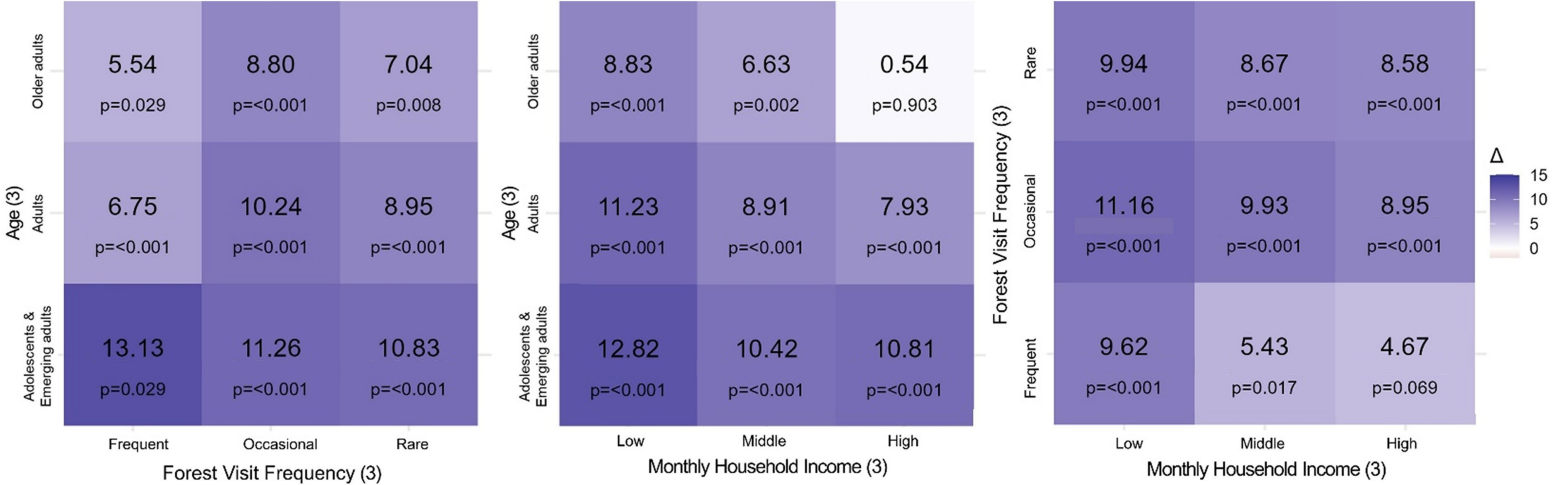
Factor-combination–specific pre–post changes.

**Figure 3:**
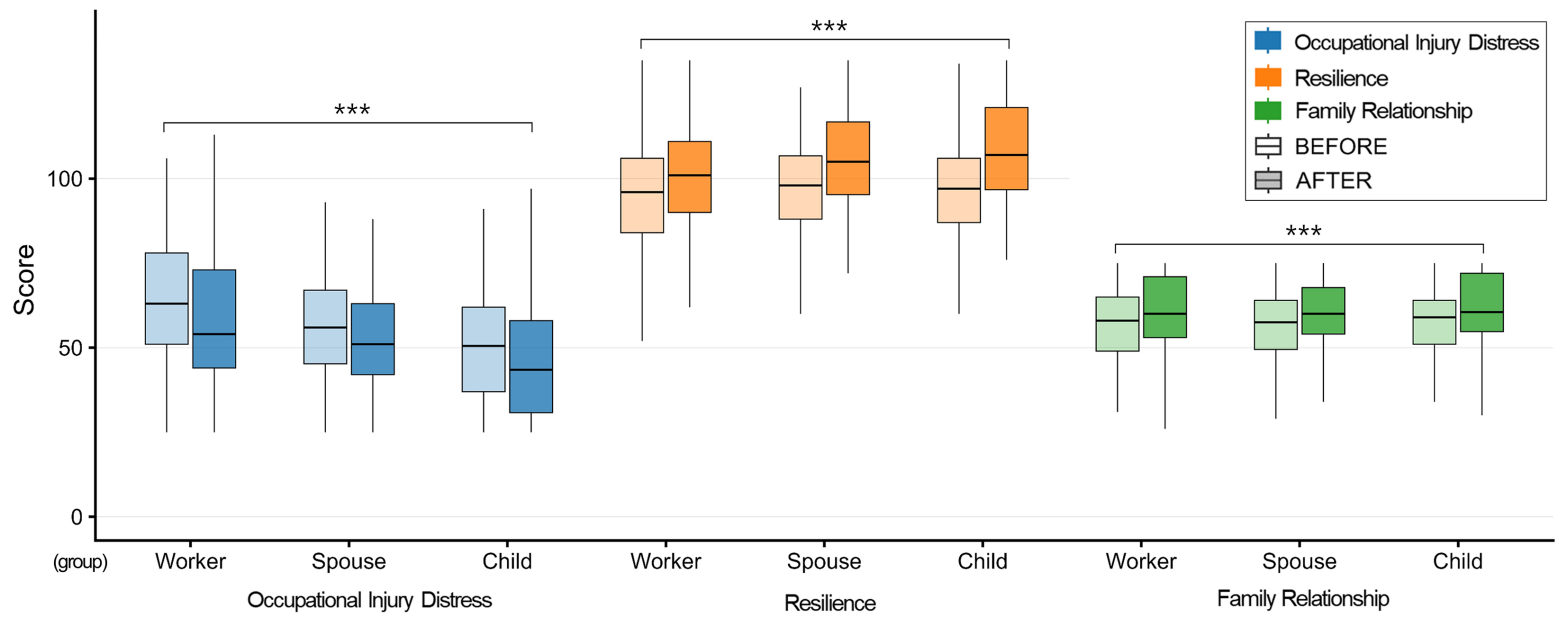
Adjusted pre-post results by family members.

A total of 1,194 participants completed both pre- and post-intervention assessments and were included in the paired analyses (Table 1, Figure 2). The sample comprised injured workers (38%), spouses (32%), children (22%), relatives (6%), and others or non-response (2%) (Table 4). Additional sociodemographic characteristics, including sex, age, household income, and frequency of forest visits, are summarized in Table 4. For the age-adjusted role-stratified linear mixed-effects models, 918 participants contributed complete data (Table 2, Figure 3).

**Table 4:** Sociodemographic characteristics of participants (*N* = 1,194).

Variable	Categories	*n*	%
Family members	Injured worker	454	38
	Family-spouse	384	32
	Family-child	259	22
	Family-relative	77	6
	Others/non-response	20	2
Gender	Male	559	47
	Female	628	52
	Non-response	7	1
Age	≤19 years	103	9
	20s	80	7
	30s	128	11
	40s	224	19
	50s	311	26
	≥60 years	323	27
	Non-response	25	2
Monthly household income (KRW)	<1 million KRW	61	5
	1–2 million KRW	71	6
	2–3 million KRW	190	16
	3–4 million KRW	249	21
	4–5 million KRW	237	20
	5–6 million KRW	0	0
	6–7 million KRW	132	11
	≥7 million KRW	111	9
Forest visit frequency	Non-response	143	12
	Daily	47	4
	1–2/week	192	16
	1–2/month	324	27
	1–2/6 month	198	17
	1–2/year	218	18
	Rarely	193	16
	Non-response	22	2

Values are presented as counts (*n*) and percentages (%). Percentages are rounded to the nearest integer; totals may not sum to 100%. Income is reported in Korean won (KRW).

Exploratory heterogeneity analyses focusing on resilience included 789 participants with complete information on age, income, and forest visit frequency (Table 3, Figure 1). To improve model stability and facilitate interpretation of interaction effects, selected sociodemographic variables were consolidated. Age was grouped into 10–29 years (≤19 and 20–29 years), 30–59 years, and ≥60 years. This categorization was informed by discussions of emerging adulthood extending into the late 20s and commonly used definitions of older adulthood at 60 years or above (37, 38).

Monthly household income was categorized as Low (<3 million KRW), Middle (3–6 million KRW), and High (≥6 million KRW), preserving ordinal structure while improving subgroup stability; these thresholds approximate the average income of a four-person household in South Korea in 2025 (39). Forest visit frequency was classified into Frequent (daily or 1–2 times per week), Occasional (1–2 times per month or per 6 months), and Rare (1–2 times per year or rarely), consistent with time-based frequency scales outlined in survey design guidelines (40).

### 3.2 Paired pre–post comparisons

Table 1, Figure 2 summarize paired pre–post comparisons for the full sample (*N* = 1,194). Across all three instruments, mean scores improved from pre to post with small-to-moderate effect sizes (Hedges’ *g*). Although formal tests indicated deviations from normality (Shapiro–Wilk *p* < 0.001 for all outcomes in Table 1), Wilcoxon signed-rank sensitivity analyses yielded consistent inferences (Supplementary Table 1). Occupational injury distress scores decreased by −7.807 (95% CI −8.787 to −6.826, *p* < 0.001, *g* = −0.397). All subscales showed significant reductions (all *p* < 0.001), with the most significant mean reduction observed for Anger (−1.843, *g* = −0.431), followed by Depression (−1.651, *g* = −0.374), Anxiety (−1.585, *g* = −0.364), Somatization (−1.572, *g* = −0.351), and Lack of Social Support (−1.161, *g* = −0.278). Resilience total scores increased by +8.508 (95% CI 7.608 to 9.409, *p* < 0.001, *g* = 0.517). Subdomain gains were comparable, including increases in Optimism (+3.111, *g* = 0.501), Self-control (+2.722, *g* = 0.469), and Sociability (+2.676, *g* = 0.467) (all *p* < 0.001). Family Relationship total scores increased by +4.376 (95% CI 3.775 to 4.977, *p* < 0.001, *g* = 0.391). Improvements were observed for Emotional Support (+3.032, *g* = 0.397) and Acceptance and Respect (+1.343, *g* = 0.342) (all *p* < 0.001). Overall, paired comparisons showed reduced psychosocial burden together with higher resilience and family relationship scores at the immediate post-program assessment.

### 3.3 Family members role-stratified adjusted pre–post effects (LMM)

Age-adjusted linear mixed-effects models (*N* = 918) indicated significant within-person pre–post changes across all instruments (Holm-adjusted *p* < 0.001 for all contrasts; Table 2). For Occupational injury distress, adjusted reductions were observed across all roles. The estimated reduction was numerically larger among injured workers (*Δ* = −9.215) than among spouses (*Δ* = −7.665) and children (*Δ* = −6.516). Thus, although improvements were evident in all groups, distress decreases appeared numerically greater among injured workers.

For resilience, adjusted increases were observed across roles, with a greater magnitude among children (*Δ* = +9.924) than among spouses (*Δ* = +8.885) and injured workers (*Δ* = +8.427). These estimates suggest that resilience increased across family members and may have been numerically larger among children.

For Family relationship quality, improvements were observed in all roles: injured workers (*Δ* = +3.754), spouses (*Δ* = +4.633), and children (*Δ* = +4.293). The increases among spouses and children were numerically greater than those observed among injured workers. This pattern may suggest that increases in perceived family relationship quality were somewhat larger among cohabiting family members, although positive changes were observed across all groups.

Overall, these adjusted analyses suggest role-differentiated patterns of change, with reductions in occupational injury distress appearing more pronounced among injured workers, resilience gains relatively greater among children, and improvements in family relationship quality observed across roles (Table 2, Figure 3).

### 3.4 Exploratory heterogeneity in pre–post change in resilience

To examine heterogeneity in resilience change, age group, forest-visit frequency, and monthly household income were categorized into three levels, forming three-level factors and analyzed using linear mixed-effects models with a subject-level random intercept (*N* = 789). These subgroup analyses were explicitly exploratory and hypothesis-generating rather than confirmatory. They were restricted to the KRQ-27 resilience outcome to reduce multiplicity burden and to focus on resilience as a theoretically central indicator of adaptive recovery.

A prespecified four-way interaction model could not be reliably estimated due to insufficient data within subgroup combinations. Therefore, three separate two-factor interaction models were fitted Post–pre changes (*Δ*) were derived from model-based estimated marginal means with Holm correction Numerical estimates are presented in Table 3 and visualized in Figure 1. Given the sparse sample sizes in some strata, these estimates should be interpreted cautiously, particularly where confidence intervals were wide and adjusted *p*-values were non-significant.

In the Visit × Age model, resilience gains were observed across visit-frequency categories. The numerically largest estimated increase was observed among adolescents and emerging adults who reported frequent forest visits (*Δ* = +13.13). However, substantial gains were also observed among occasional and rare visitors in this age group. Across visit categories, younger participants tended to show larger estimated increases than older adults; changes among older adults were generally minor in magnitude.

In the Income × Age model, improvements were observed across nearly all strata. The numerically largest estimated increase was observed among adolescents and emerging adults in the low-income group (*Δ* = +12.82), followed by adults in the same income category (*Δ* = +11.23). Across income levels, younger participants consistently demonstrated greater estimated gains relative to older adults. In contrast, older adults in the high-income group showed minimal estimated change, accompanied by wide confidence intervals and non-significant adjusted *p*-values, suggesting greater statistical uncertainty in this subgroup.

In the Income × Visit model, improvements were observed in most income-by-visit combinations. The largest estimated increase was observed among low-income participants reporting occasional visits (*Δ* = +11.16). Positive changes were also evident among low-income rare visitors (*Δ* = +9.93) and middle-income occasional visitors (*Δ* = +9.93). In contrast, high-income participants with frequent visits showed no significant change, with comparatively wide confidence intervals.

Across interaction models, resilience gains were broadly observed across demographic and behavioral strata. Numerically larger improvements tended to appear among younger participants and among low-income groups in several combinations. However, because these subgroup analyses were exploratory, involved multiple testing, and included sparse cell sizes in some strata, the apparent differences should be interpreted with substantial caution. These findings are best understood as hypothesis-generating patterns that may inform future confirmatory analyses, rather than as evidence of robust subgroup-specific effects.

## 4 Discussion

This study evaluated a forest-based intervention delivered to occupationally injured workers and their cohabiting family members. Although delivered within designated therapeutic forest environments, the intervention was embedded within broader urban and peri-urban greenspace systems accessible to metropolitan households. Therefore, the findings may contribute to the growing literature on urban greening as a nature-based approach within rehabilitation contexts. Within the broader context of urban greening, structured exposure to accessible green spaces may represent one supportive element within health-oriented environments (41).

In the full sample (*N* = 1,194), pre–post comparisons showed statistically significant reductions in psychological burden and increases in resilience and perceived family relationship quality. In age-adjusted linear mixed-effects models (*N* = 918), changes differed across family roles. Injured workers showed greater reductions in occupational injury distress than spouses or children. This may reflect higher baseline distress among workers, leading to larger observable decreases. Although theoretical models of forest-based interventions emphasize mechanisms such as attentional restoration and autonomic regulation (42, 43), the study collected no physiological indicators; therefore, mechanistic interpretations remain tentative.

Resilience increased across all roles, with larger numerical gains among children and spouses than among injured workers. Family relationship quality also improved across roles and appeared more pronounced among spouses and children. These findings suggest that the intervention may have been associated with improvements in both individual psychological outcomes and perceived family functioning. Occupational injury often functions as a sustained stressor at the household level, and supportive environments may facilitate adaptive responses (44). However, given the single-group pre-post design, interpretations should remain cautious.

Exploratory heterogeneity analyses focused on resilience (*N* = 789). Across most age, income, and forest-visit strata, resilience increased from pre to post. Some interaction patterns suggested greater improvements among younger participants and among lower- and middle-income groups. In urban greening research, differential responsiveness across socioeconomic strata has been discussed in relation to environmental equity (10, 45). Nevertheless, several subgroup combinations were small, and these analyses were exploratory; therefore, findings should be interpreted cautiously.

Recruiting entire households enabled a direct comparison of role-specific responses within a shared greenspace-based intervention. The present findings suggest that forest-based programs situated within urban or peri-urban greenspace systems may be associated with lower distress scores and higher perceived household cohesion. Among injured workers, reductions in psychosocial burden appeared numerically larger, whereas among spouses and children, gains in resilience and perceived relationship quality appeared numerically larger. Although the observed effect sizes were generally in the small-to-moderate range, their practical relevance, if any, may lie in the fact that these changes were detected over a short intervention period in a population experiencing occupational injury and related family strain. In this context, even modest short-term changes in distress, resilience, and perceived family functioning may be relevant as provisional indicators of early psychosocial stabilization. At the same time, these differences should not be overstated as evidence of clinically established benefit, particularly in the absence of longer-term follow-up or external clinical anchors. Rather, the present findings may be understood as broadly consistent with prior forest-therapy and nature-based intervention literature reporting modest short-term improvements in psychological well-being, while extending that literature to an occupational-injury household context.

This investigation has several strengths and limitations. Strengths include the inclusion of multiple family roles within a single intervention framework and the use of mixed-effects modeling to estimate adjusted change. More than 1,000 participants contributed to the paired analyses, representing a relatively large cohort for a household-level greenspace intervention study. At the same time, the present findings should be interpreted with appropriate caution. Because this study used a single-group pre–post design without a control condition, the observed improvements cannot be interpreted as confirmed causal effects of the intervention, but rather as short-term associations observed after participation in the program. In addition to the intervention itself, alternative explanations such as regression to the mean, temporary relief from distressing circumstances, supportive social interaction during the program, or other non-specific effects related to study participation may also have contributed to the observed pre–post changes.

Further limitations include the lack of long-term follow-up, reliance on self-report measures, and the absence of household-level identifiers in the de-identified analytic dataset, which meant that household-level random effects could not be incorporated into the mixed models. Although the program was delivered using a standardized module by nationally certified forest therapy practitioners and session checklists were used to monitor adherence, some variation in implementation may still have occurred across sites, facilitators, and sessions. Such variability in delivery style, emphasis of activities, or local program context could have contributed to heterogeneity in observed pre–post changes. Because quantitative fidelity indicators were not available in the analytic dataset, the present study could not directly evaluate the extent to which implementation differences influenced outcomes. Furthermore, the present analyses did not distinguish participants by injury severity, chronicity, functional limitation, or newly acquired disability status; this limits the precision with which the findings can be interpreted across heterogeneous injured-worker populations. Some subgroup estimates were also imprecise because several interaction strata were sparse. Furthermore, because outcomes were assessed immediately after program completion within a brief assessment window, the present results do not establish sustained benefit over longer follow-up periods. As a result, some residual within-household dependence may remain unaccounted for, and model precision should be interpreted with caution.

In summary, forest-based interventions embedded within urban greenspace systems were associated with short-term favorable changes in psychological and relational outcomes among households affected by occupational injury. Future studies, particularly controlled and longitudinal designs that include physiological indicators, are needed to understand better how these interventions work.

## 5 Conclusion

This mixed-effects pre–post study identified statistically significant short-term changes across three domains: lower psychological burden, higher resilience, and higher perceived family relationship quality at the immediate post-program assessment. Participants reported decreases in anger, anxiety, depressive symptoms, somatization, and perceived lack of social support, alongside increases in optimism, self-regulation, interpersonal competence, emotional support, and mutual respect. These findings suggest that participation in a brief forest-based intervention was associated with favorable changes in both individual psychological outcomes and perceived relational functioning among households affected by occupational injury.

Role-stratified linear mixed-effects models suggested differences in the magnitude of change across family members. Injured workers showed numerically larger reductions in psychological burden, whereas children and spouses showed numerically larger gains in resilience. Perceived family relationship quality increased across all roles. Exploratory interaction analyses suggested that resilience gains were more evident in lower- and middle-income strata; however, some subgroup combinations did not remain statistically significant after multiplicity adjustment, and these patterns should therefore be interpreted cautiously.

Overall, the findings suggest that household-level forest-based programming embedded within urban greenspace systems may be associated with short-term favorable psychological and relational changes in families recovering from occupational injury. Because the study employed a single-group pre–post design without a control condition and assessed only immediate post-intervention outcomes, causal inferences cannot be drawn, and the durability of effects remains unknown. Future research incorporating controlled designs, longer follow-up periods, and objective physiological indicators will be necessary to clarify mechanisms and to determine the long-term relevance of such interventions within rehabilitation systems.

## Data Availability

The datasets generated and analyzed during the current study are not publicly available due to privacy protection regulations and institutional data governance policies. Requests to access the datasets should be directed to the corresponding author and are subject to approval by the Korea Forest Welfare Institute (FoWI).
