# Supplementary material for: Household-level surrounding greenspace as a nature-based intervention for health recovery after occupational injury
Source: Front Public Health. 2026 May 1;14:1817203. doi: 10.3389/fpubh.2026.1817203 (PMC13176137; doi:10.3389/fpubh.2026.1817203)
Supplement: Supplementary file 1 [file Data_Sheet_1.PDF]

Supplementary Material

Supplementary Table1. Wilcoxon signed-rank test for Occupational Injury Distress, Resilience, Family Relationship

	Scale	Wilcoxon W	Hodges-Lehmann shift (After-Before)	95% CI (HL)	*p*	*p* (Holm)	*p* (BH)
Occupational Injury Distress	Anger	102005.500	-2.000	[-2.500, -2.000]	< .001	< .001	< .001
	Anxiety	118463.000	-1.500	[-2.000, -1.500]	< .001	< .001	< .001
	Depression	110261.000	-2.000	[-2.000, -1.500]	< .001	< .001	< .001
	Somatization	126631.500	-1.500	[-2.000, -1.500]	< .001	< .001	< .001
	Lack of Social Support	130598.500	-1.000	[-1.500, -1.000]	< .001	< .001	< .001
	TOTAL	138725.000	-6.500	[-8.000, -6.000]	< .001	< .001	< .001
Resilience	Self-control	454793.500	3.000	[2.500, 3.000]	< .001	< .001	< .001
	Sociability	432299.500	3.000	[2.500, 3.000]	< .001	< .001	< .001
	Optimism	438628.000	3.500	[3.000, 3.500]	< .001	< .001	< .001
	TOTAL	535673.000	8.000	[7.000, 8.500]	< .001	< .001	< .001
Family Relationship	Emotional Support	392864.500	3.500	[3.000, 4.000]	< .001	< .001	< .001
	Acceptance & Respect	277323.500	2.000	[1.500, 2.000]	< .001	< .001	< .001
	TOTAL	408151.500	5.000	[4.000, 5.500]	< .001	< .001	< .001

Notes. N=1194. Hodges-Lehmann shift is the estimated median of paired differences with 95%. Holm and Benjamini-Hochberg corrections were applied for multiple comparisons.

Supplementary Table 2. Internal consistency (Cronbach's α) by set.

	Set	α Before	α After
Occupational Injury Distress	Anger	0.855	0.873
	Anxiety	0.875	0.890
	Depression	0.893	0.905
	Somatization	0.738	0.857
	Lack of Social Support	0.886	0.897
Resilience	Self-control	0.890	0.922
	Sociability	0.871	0.918
	Optimism	0.879	0.915
Family Relationship	Emotional Support	0.952	0.951
	Acceptance & Respect	0.933	0.949

Supplementary Table 3. Forest intervention program schedule

<table>
<tr><th>Time</th><th>Day 1</th><th>Day 2</th></tr>
<tr><td>08:00-09:00</td><td rowspan="4">Arrival</td><td>Breakfast</td></tr>
<tr><td>09:00-10:00</td><td>Break</td></tr>
<tr><td>10:00-11:00</td><td rowspan="2">Educational Program: Family-oriented forest interpretation, experience, and play</td></tr>
<tr><td>11:00-12:00</td></tr>
<tr><td>12:00-13:00</td><td>Lunch</td><td>Lunch</td></tr>
<tr><td>13:00-14:00</td><td>Orientation and pre-program effectiveness scale assessment</td><td>Transfer and break</td></tr>
<tr><td>14:00-15:00</td><td rowspan="2">Educational Program:
Forest mission orienteering</td><td>Regional Linkage Program: Institution-specific specialized program</td></tr>
<tr><td>15:00-16:00</td><td>Post-program effectiveness scale assessment</td></tr>
<tr><td>16:00-17:00</td><td rowspan="2">Physical Activity Program: Stretching with small equipment and resistance bands</td><td rowspan="3">Departure</td></tr>
<tr><td>17:00-18:00</td></tr>
<tr><td>18:00-19:00</td><td>Dinner</td></tr>
</table>

Notes. The table presents an example of the structured 1-night/2-day program schedule used in the participating forest welfare facilities. Program timing and institution-specific regional linkage activities could vary slightly across sites, but the overall sequence of orientation, baseline assessment, guided forest-based activities, and immediate post-program assessment was maintained.
